# Continuum of care approach for managing non-communicable diseases in low- and middle-income countries

**DOI:** 10.7189/jogh.10.010337

**Published:** 2020-06

**Authors:** Rajshree Thapa, Ayse Zengin, Amanda G Thrift

**Affiliations:** Department of Medicine, School of Clinical Sciences at Monash Health, Monash University, Melbourne, Australia

*The Lancet* report on the non-communicable diseases (NCDs) countdown reveals that the current progress is sub-optimal. Indeed, at current rates of decline, 86 countries (46%) for women and 97 countries (52%) for men, will fail to achieve the sustainable development goal (SDG) target by 2030 [1]. High income countries (HICs) have been successful in reducing mortality from NCDs (cardiovascular disease, diabetes, cancer and chronic respiratory disease), although mainly from reductions in cardiovascular deaths. This decline in cardiovascular mortality has been achieved by a rapid progress in both prevention and treatment, including declines in cigarette smoking, improvements in controlling hypertension, and use of statins, thrombolysis and stents [2]. Unfortunately, cardiovascular deaths, which form the largest proportion of deaths from NCD, are not declining in most Sub-Saharan and Central Asian countries [3] and there are instances where such mortality has even increased in as many as 15 countries for women and 24 countries for men [1].

**Figure 1:**
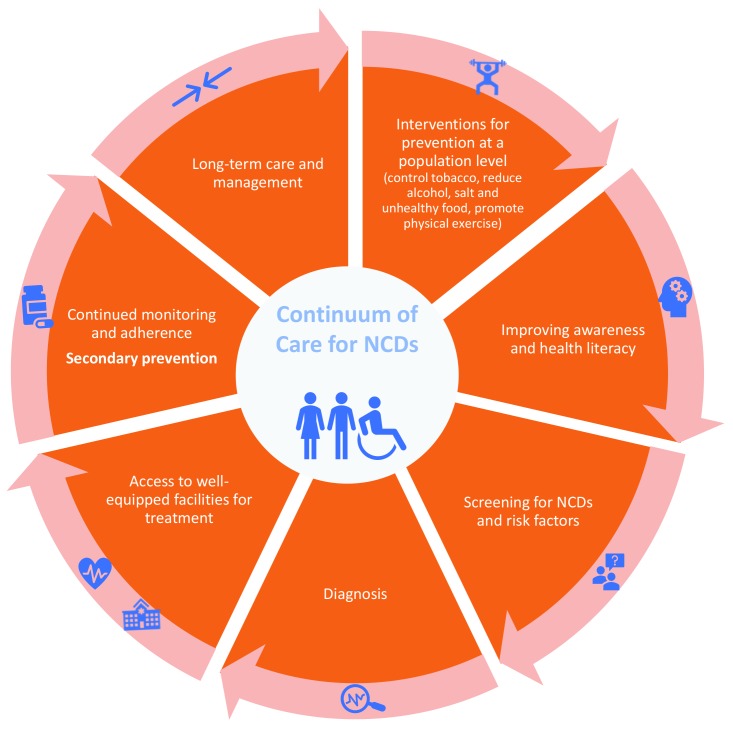
Continuum of care for the management of NCDs. Adapted from [4] with permission.

Collaborative efforts are required to curb the NCD burden and mortality in these countries. A continuum of care approach is an important public health tool that can provide crucial understanding into all stages of disease progression, from prevention through to long-term care, and would enable consolidation of efforts to control NCDs (**Figure 1**). While the model has been widely used in managing chronic conditions such as HIV [5], it has not been optimally used to monitor the impact across the various spectra of NCD care.

There is already compelling evidence for targeting services across the continuum of care for NCDs: prevention, diagnosis, treatment and long-term care. Ford et al. reported that evidence-based medical and surgical treatments reduced cardiovascular deaths deaths in USA by 47%, while reductions in major risk factors reduced cardiovascular deaths by 44% [6]. Owolabi proposed a stroke quadrangle comprising primary and secondary prevention, access to well-equipped care and rehabilitation and research networks to address the burden of stroke in Africa [7]. Countries in Sub-Saharan Africa, Central and South Asia still face a challenge across the continuum as routine health check-ups and/or long-term follow up care are not part of the regular health system. Similarly, poverty and other social determinants of health is contributing to poor access to diagnosis and treatment of chronic conditions in Sub-Saharan Africa [3].

Implementation of NCD prevention policies and adoption of the WHO “best buy” interventions varies by country within, and across, the region. There are still gaps in terms of implementation of the framework convention for the tobacco control (FCTC) and policies to curb the harmful use of alcohol, as reported in five African countries [8]. Implementation of policy interventions for both diet and physical activity have been less prioritized. In most instances, there are no population level data enabling evaluation of these population level interventions to strengthen the strategies [9].

Low-and middle-income countries (LMICs) face a major barrier in accessing diagnosis, treatment and care; in countries such as Bhutan and Nepal, more than 60% of adults with hypertension remain unaware of their condition [10]. Similarly, more than half of the cohort with hypertension in the HealthRise project in South Africa were unaware of their status [4]. Among those aware, a significant proportion never accessed treatment or were otherwise lost to follow-up [4]. Thus, there remains a wide gap in achieving treatment and control of hypertension.

Many countries have inadequate health services to support NCD management. In Tanzania, health facilities at all levels appear ill prepared to address the diagnosis, treatment, and ongoing monitoring needs of patients with NCDs [11]. Specific to hypertension, in a survey of health facilities in Nepal, not all facilities had digital or manual blood pressure devices [12]. Only about one in five health facilities offered services for the diagnosis and/or management of diabetes with less than a quarter of those sites, having stock of essential anti-diabetic medicines [12]. The declining rate of mortality in HICs is attributed to their effective prevention strategies, prompt diagnosis, referral services and long-term care services that are lacking in many LMICs. Successful implementation of prevention strategies for NCDs lie within the political will of countries to curb alcohol, tobacco use, discourage unhealthy foods and drinks, and promote physical activity. Equally important is strengthening the health system to respond to treatment needs and long-term monitoring to achieve control of risk factors. Improving adherence through patient education and communication, improving availability and affordability of medication and continuous monitoring and follow up is critical for the control of hypertension and other cardiovascular deaths [3]. Transferring tasks to community health volunteers, who have primarily been active in controlling infectious diseases and improving maternal and child health, may potentially be effective in improving diagnosis, monitoring and adherence to treatment for NCDs in LMICs [13].

**Figure:**
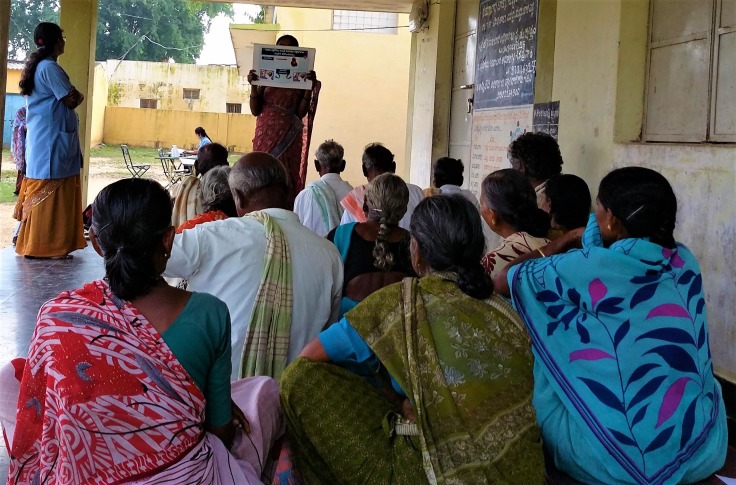
Photo: From the collection of Oduru Suresh, used with permission.

Single fragmented approaches to achieve the SDG goals are not working. Strategies must be strengthened to improve outcomes across the NCD continuum, from prevention through to long-term care. This must include a robust monitoring system, with indicators set for each process in the pathway of care. Without this, we will not achieve the target to reduce NCD related mortality by one third by 2030.
